# Iodide of Potassium

**Published:** 1841-11

**Authors:** James C. L. Carson


*Iodide of Potassium.* By James C. L. Carson, M. D.—The perusal of the article "Iodide of Potassium," in Pereira's Materia Medica, brings to my recollection a case which occurred in my practice about three years ago. I ordered a gentleman three grains of iodide of potassium in a draught of peppermint water, three times a day. When he had taken the medicine three times he felt poorly; and in the course of an hour after the fourth dose he was attacked with a violent shivering fit, followed by intense headache, heat of skin, constant thirst, quick and very full pulse, and vomiting and purging at the same time. These symptoms were succeeded by great prostration of strength. Notwithstanding the exhibition of demulcents and opiates the purging lasted for several days. The effects of the medicine in this case were so violent that I have little doubt, that if he had taken another dose, his life would have been forfeited. This is the only instance, which I have seen, of the iodide of potassium producing unpleasant effects in doses under ten grains.—*Ibid.*

